# Author Correction: Multimodal analysis of cfDNA methylomes for early detecting esophageal squamous cell carcinoma and precancerous lesions

**DOI:** 10.1038/s41467-026-75462-2

**Published:** 2026-08-25

**Authors:** Jiaqi Liu, Lijun Dai, Qiang Wang, Chenghao Li, Zhichao Liu, Tongyang Gong, Hengyi Xu, Ziqi Jia, Wanyuan Sun, Xinyu Wang, Minyi Lu, Tongxuan Shang, Ning Zhao, Jiahui Cai, Zhigang Li, Hongyan Chen, Jianzhong Su, Zhihua Liu

**Affiliations:** 1 State Key Laboratory of Molecular Oncology, National Cancer Center/National Clinical Research Center for Cancer/Cancer Hospital, Chinese Academy of Medical Sciences and Peking Union Medical College, Beijing, China; 2 Department of Breast Surgical Oncology, National Cancer Center/National Clinical Research Center for Cancer/Cancer Hospital, Chinese Academy of Medical Sciences and Peking Union Medical College, Beijing, China; 3 Oujiang Laboratory (Zhejiang Lab for Regenerative Medicine, Vision and Brain Health), Eye Hospital, Wenzhou Medical University, Wenzhou, China; 4 Department of Anesthesiology, National Cancer Center/National Clinical Research Center for Cancer/Cancer Hospital, Chinese Academy of Medical Sciences and Peking Union Medical College, Beijing, China; 5 Department of Thoracic Surgery, Shanghai Chest Hospital, Shanghai Jiao Tong University School of Medicine, Shanghai, China

**Keywords:** Tumour biomarkers, Oesophageal cancer, DNA methylation

Correction to: *Nature Communications* 10.1038/s41467-024-47886-1, published online 2 May 2024

In the version of this article initially published, there was an error in Fig. 2a, Supplementary Fig. 3a, and the corresponding Source Data files, in which the cfDNA malignant ratios for sample ‘EC_1’ were inaccurate. All erroneous data entries have been removed and replaced with the correct values in a corrected version of the Source Data files. The figures and source data have been updated in the HTML and PDF versions of the article.

## Supplementary information

Incorrect Fig. 2

Correct Fig. 2

