# Supplementary material for: Multimodal analysis of cfDNA methylomes for early detecting esophageal squamous cell carcinoma and precancerous lesions
Source: Nat Commun. 2024 May 2;15:3700. doi: 10.1038/s41467-024-47886-1 (PMC11065998; doi:10.1038/s41467-024-47886-1)
Supplement: Supplementary file 1 — Supplementary Information [file 41467_2024_47886_MOESM1_ESM.pdf]

Supplementary Information

Liu *et al.* Multimodal analysis of cfDNA methylomes for early detecting esophageal squamous cell carcinoma and precancerous lesions.

Contents

Contents 1

Supplementary Figure 1. Plasma cfDNA concentrations, sequencing coverage, and selection of the optimal number of cfDNA methylation markers. 2

Supplementary Figure 2. Cell-free DNA methylation markers selection. 3

Supplementary Figure 3. The malignant ratios of the optimal differentially methylated regions and the potential biological significance of the functional genes within them in early-stage ESCC.. 4

Supplementary Figure 4. Performances of short fragment ratios in 83 bins. 6

Supplementary Figure 5. Complementarities of the three cfDNA features. 7

Supplementary Figure 6. The specificity of the DNA methylation markers in the ESCC-cfMeth model across cell types. 9

Supplementary Figure 7. The overall survival of the ESCC patients in methylation-dominate group and the methylation-moderate/poor groups. 10

Supplementary Figure 8. The molecular and clinical characteristics of the methylation-dominate, methylation-moderate, and methylation-poor groups. 11

Supplementary Table 1. Information of the participants. 12

Supplementary Table 2. Performance of ESCC-cfMeth model. 13

Supplementary Table 3. Performance of combined models. 14

Supplementary Table 4. The overlapping of the *TP53* and APOBEC genes and the ESCC cfDNA markers. 15

Reference 15

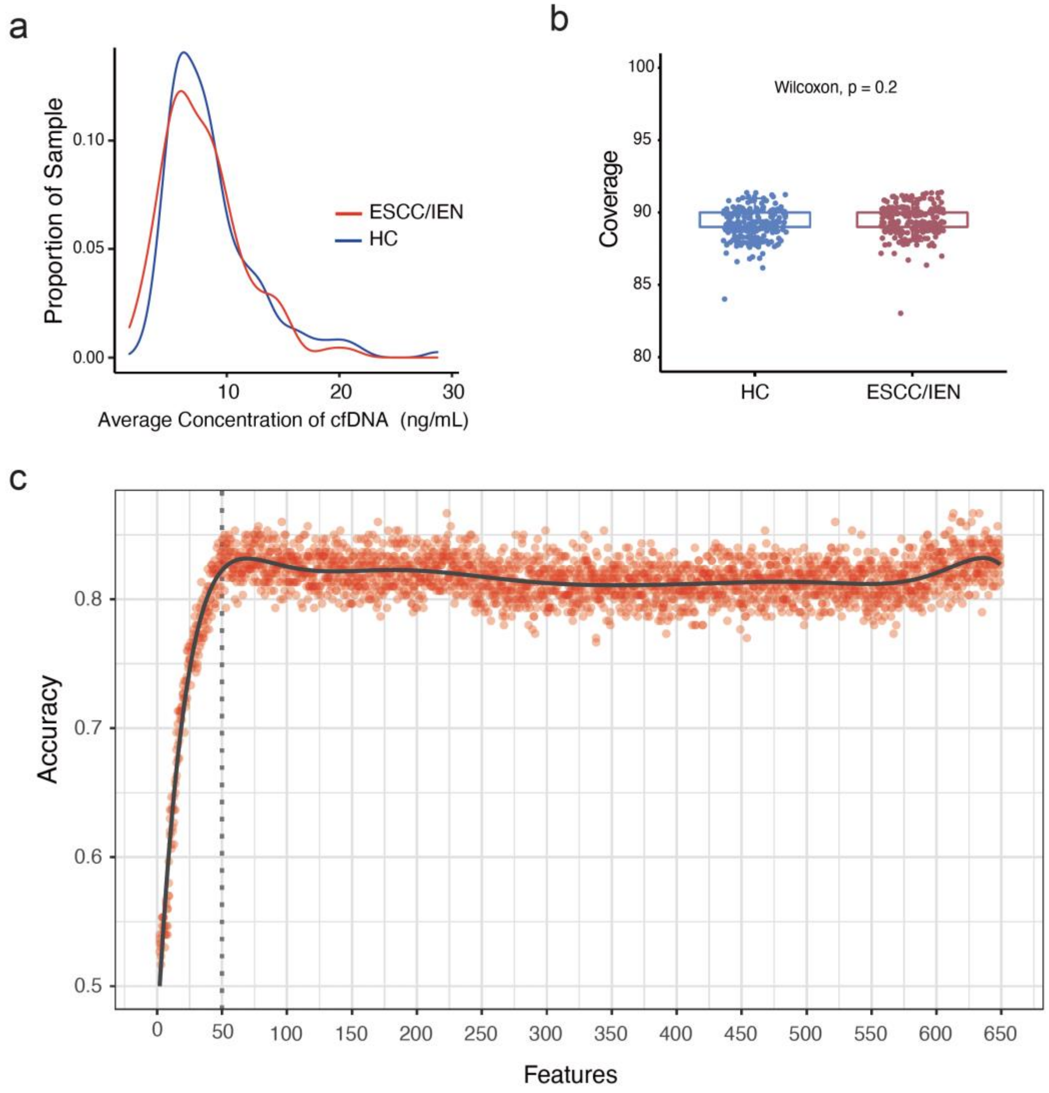

Supplementary Figure 1. Plasma cfDNA concentrations, sequencing coverage, and selection of the optimal number of cfDNA methylation markers.

a. No significant difference was found in the cfDNA concentrations between the patients with esophageal squamous cell carcinoma (ESCC) and healthy controls (HCs). **b.** The whole-genome bisulfite sequencing (WGBS) data of cfDNA covered 89% of the reference genome on average with 9.51× depth. Data are presented as median values with maximums and minimums. **c.** Using data from the discovery cohort, a random forest algorithm was adopted to generate prediction models using the cfDNA malignant ratios of the top 1 to 650 differentially methylated regions (DMRs). The top 50 DMRs achieved the optimal performance for distinguishing between malignant and benign plasma samples. Abbreviation: ESCC, esophageal squamous cell carcinoma; IEN, intraepithelial neoplasia; HC, healthy control. Source data are provided as a Source Data file.

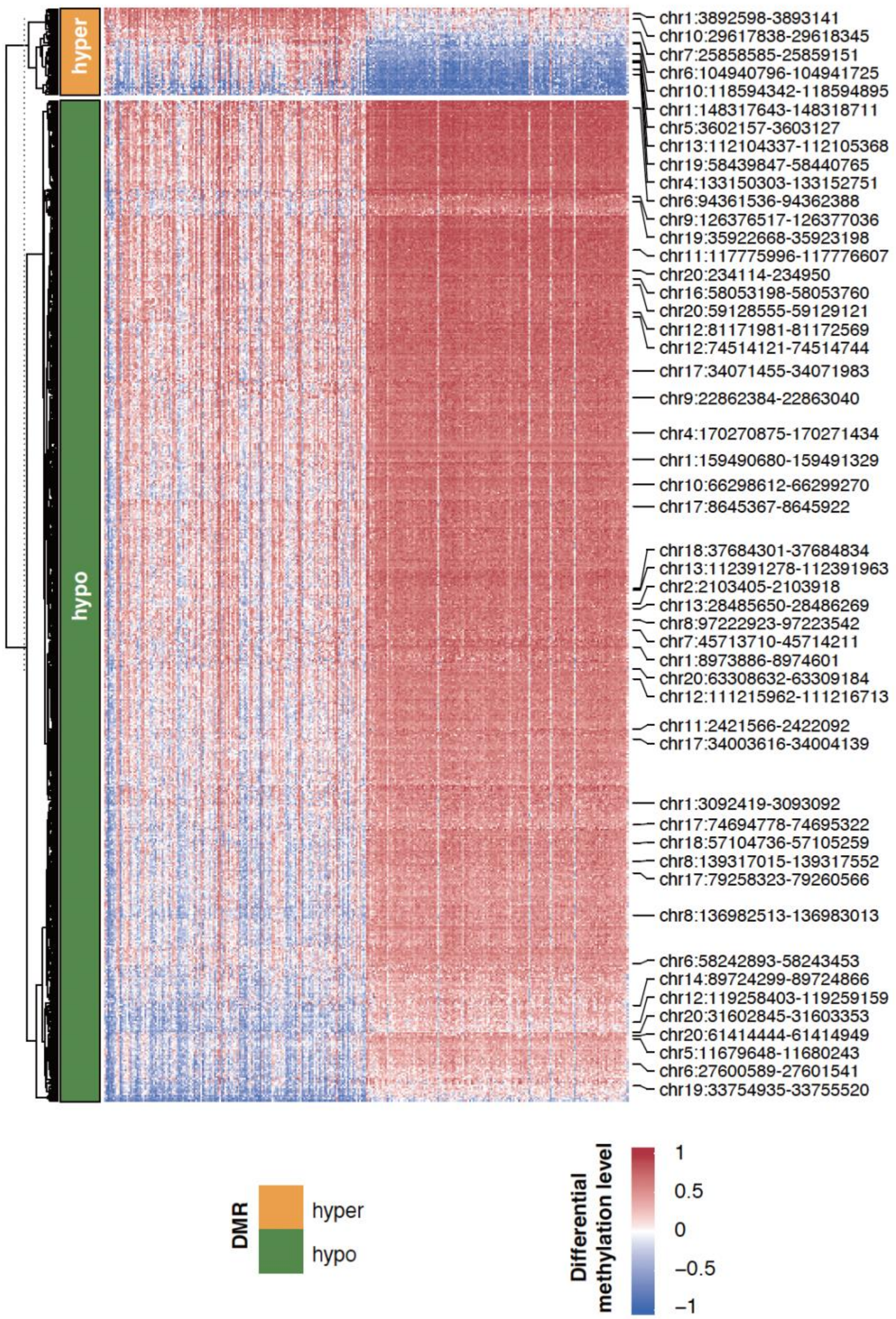

Supplementary Figure 2. Cell-free DNA methylation markers selection.

Among the 650 differentially methylated regions (DMRs) of esophageal squamous cell carcinoma (ESCC) cfDNA in the discovery cohort, the optimal 50 DMRs included 40 hypo-DMRs and 10 hyper-DMRs. Abbreviation: DMR, differentially methylated region; hypo, hypomethylation; hyper, hypermethylation. Source data are provided as a Source Data file.

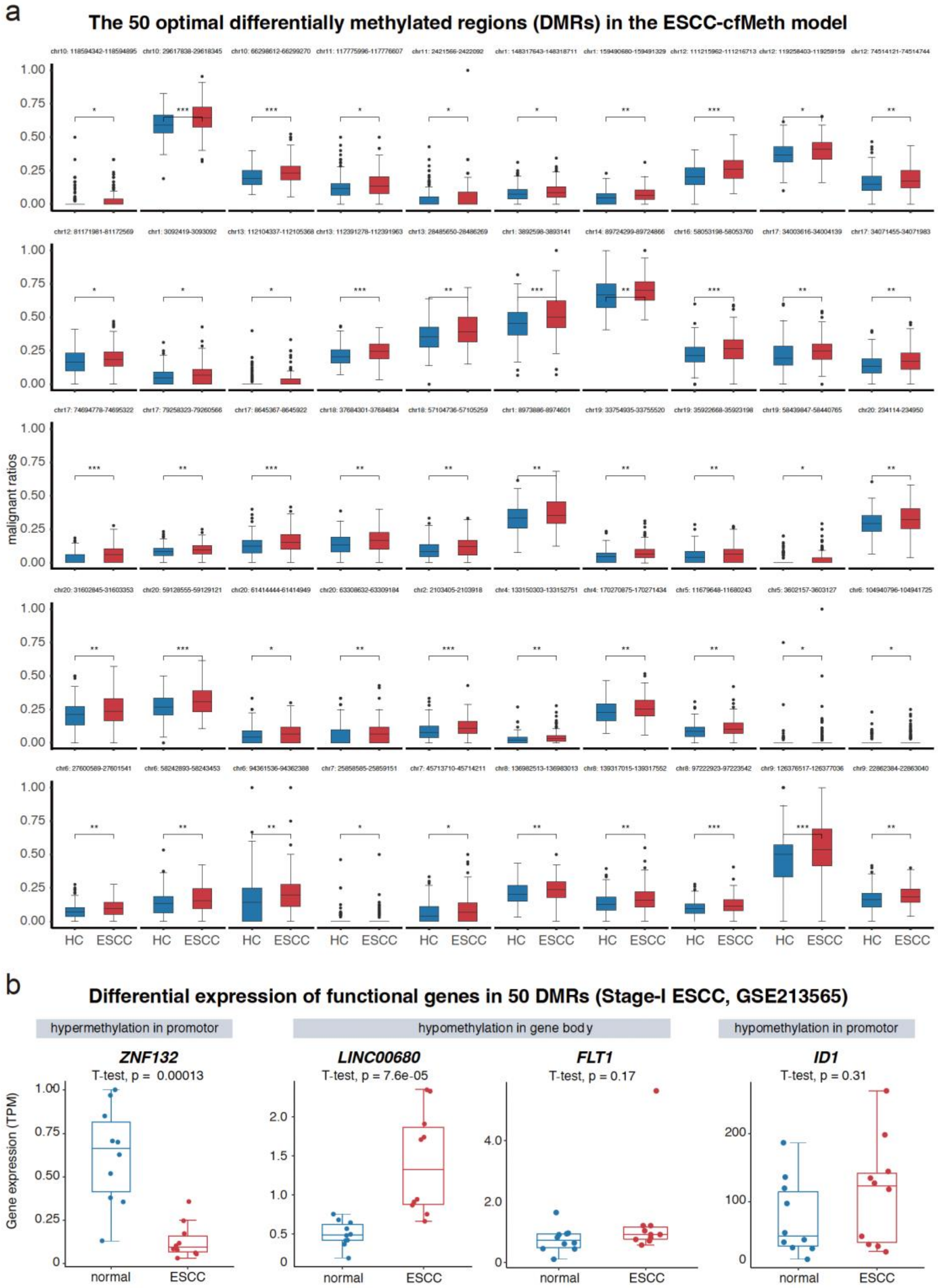

Supplementary Figure 3. The malignant ratios of the optimal differentially methylated regions and the potential biological significance of the functional genes within them in early-stage ESCC.

a. The 50 optimal differentially methylated regions (DMRs) in the ESCC-cfMeth model. The malignant ratios in these regions were significantly different between the ESCC patients and healthy controls in the discovery cohort. Data are presented as median values with maximums and minimums. * $p \leq 0.05$; ** $p \leq 0.01$; *** $p \leq 0.001$. **b.** To reveal the biological significance of the functional genes within the 50 optimal DMRs in early-stage ESCC, we analyzed the expression levels of these genes in 10-pair tissue samples of stage-I ESCC and normal tissues from a published dataset [GSE213565]. *ZNF132* with a hypermethylated promoter displayed significant down-regulation, and *LINC00680* with hypomethylation within its gene body showed upregulation. In addition, although there is no statistical significance, *FLT1* and *ID1* were also up-regulated. Data are presented as median values with maximums and minimums. Abbreviation: ESCC, esophageal squamous cell carcinoma; HC, healthy control; TPM, transcripts per million. Source data are provided as a Source Data file.

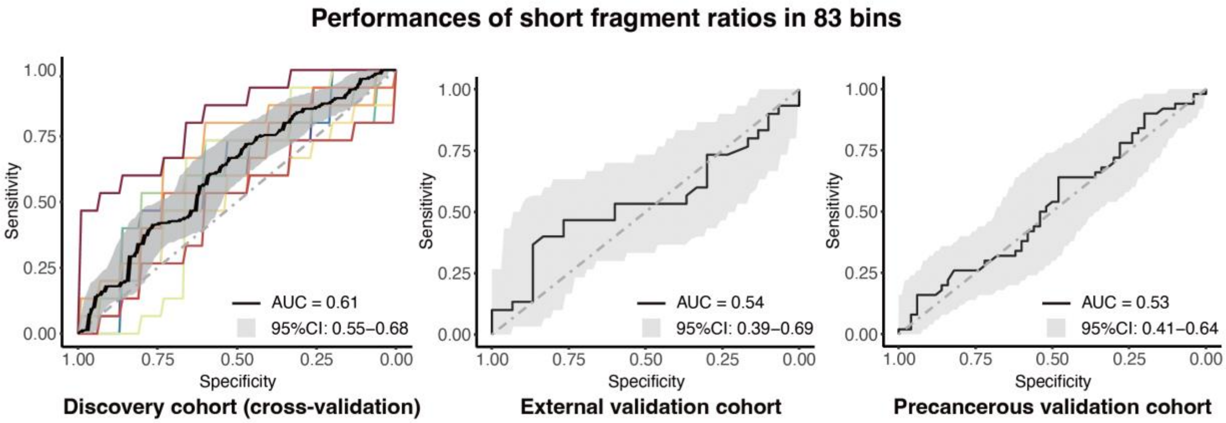

Supplementary Figure 4. Performances of short fragment ratios in 83 bins.

The diagnostic performances of the fragment size ratios (FSRs) in the 83 selected regions where FSRs were significantly elevated in ESCC patients than HCs in the discovery cohort were evaluated in the discovery cohort (10-fold cross-validation), the external validation cohort, and the precancerous validation cohort. Abbreviation: ESCC, esophageal squamous cell carcinoma; HC, healthy control; AUC, area under curve; CI, confidence interval. Source data are provided as a Source Data file.

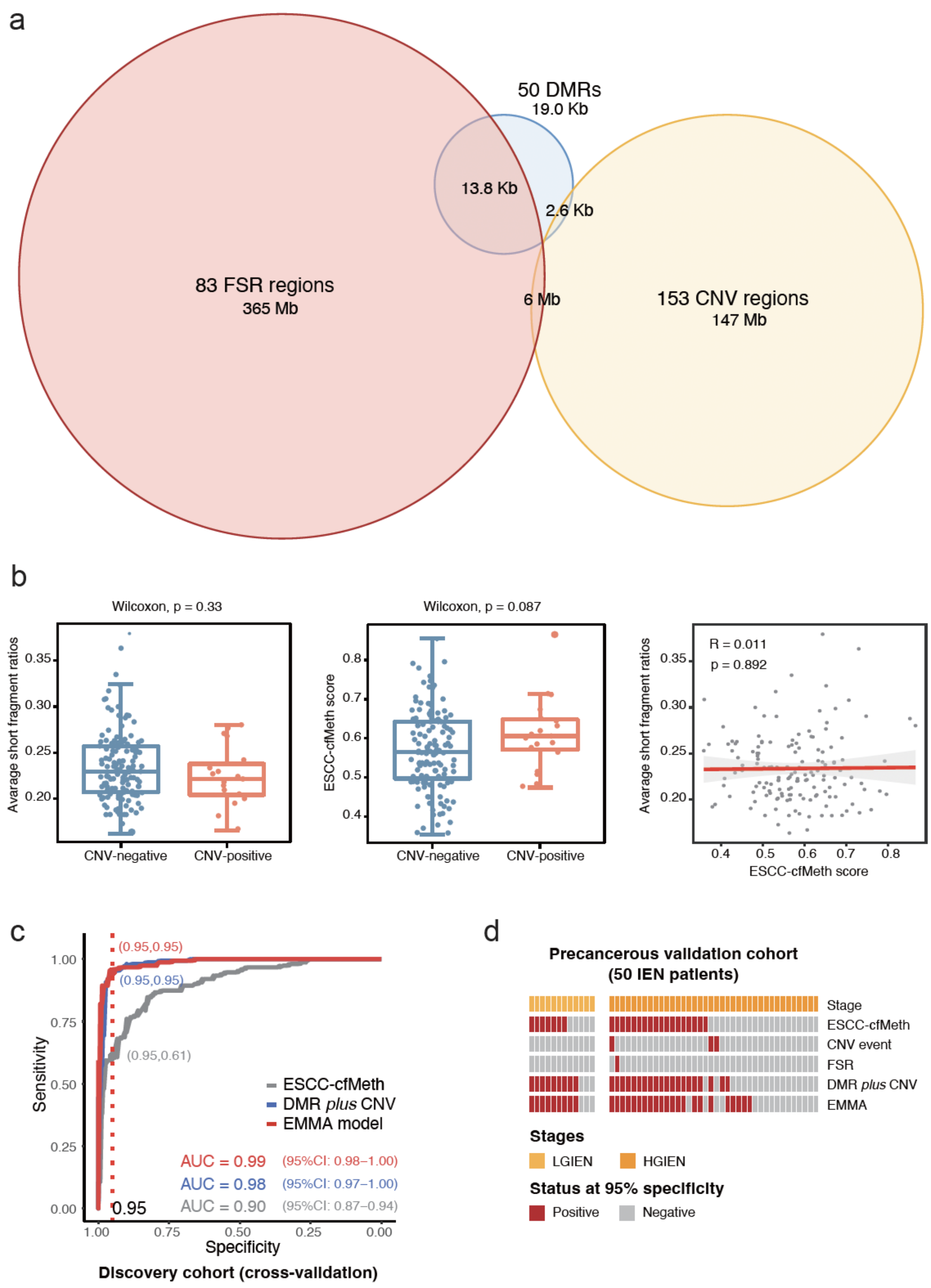

Supplementary Figure 5. Complementarities of the three cfDNA features.

a. The overlapping of the cfDNA methylation markers (differentially methylated regions, DMRs), copy number variants, and fragmentation features in the human genome. **b.** There was no significant association between the ESCC-cfMeth score, average fragment size ratio, and the copy number variant (CNV) events in the discovery cohort. Data are presented as median values with maximums and minimums. **c.** The diagnostic performances of the DMR *plus* CNV and EMMA models were evaluated in the discovery cohort (10-fold cross-validation). **d.** In the precancerous validation cohort, improved performances of the combined models resulted from the complementarities in three features. Notably, The EMMA model detected additional patients with intraepithelial neoplasia which were negative in all three single-modal models. Abbreviation: DMR, differentially methylated region; FSR, fragment size ratio; CNV, copy number variant; AUC, area under curve; CI, confidence interval; IEN, intraepithelial neoplasia; LGIEN, low-grade IEN; HGIEN, high-grade IEN; EMMA, expanded multimodal analysis. Source data are provided as a Source Data file.

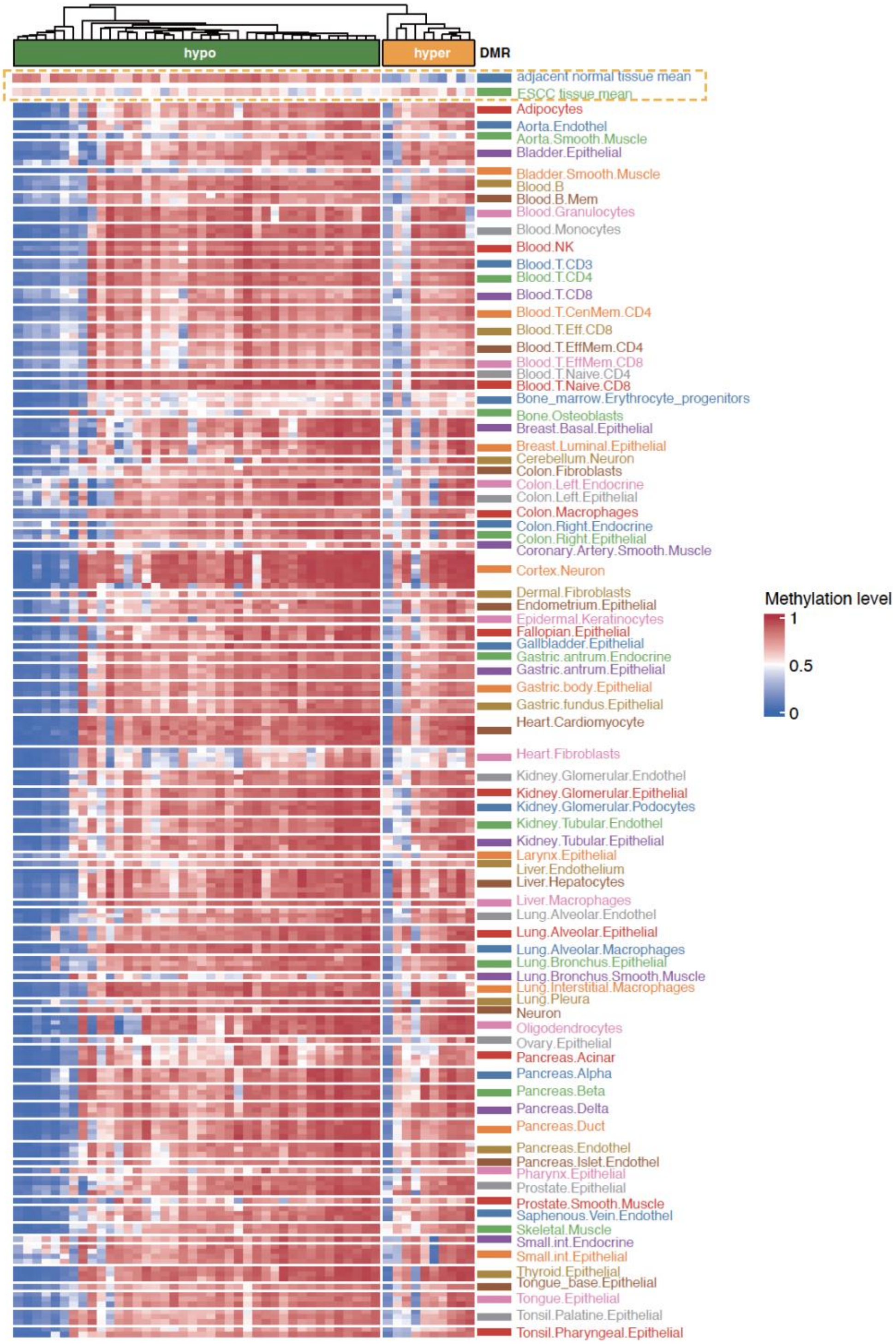

Supplementary Figure 6. The specificity of the DNA methylation markers in the ESCC-cfMeth model across cell types.

The methylation level of the 50 differentially methylated regions of the ESCC-cfMeth model in 81 common cell types[1] was analyzed and compared to the primary tumors of ESCC and matched

adjacent nonneoplastic tissues from the ECGEA cohort[2]. Abbreviation: ESCC, esophageal squamous cell carcinoma; DMR, differentially methylated region; hypo, hypomethylation; hyper, hypermethylation; ECGEA, the ESCC Genome and Epigenome Atlas. Source data are provided as a Source Data file.

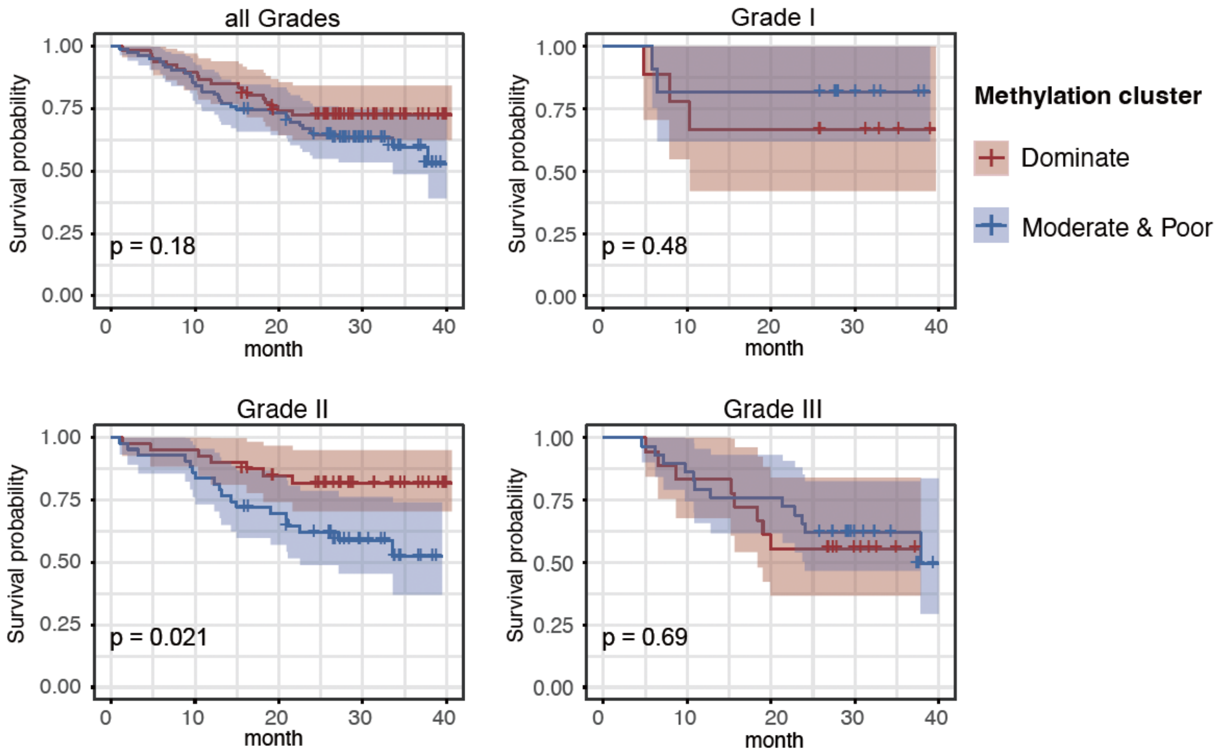

Supplementary Figure 7. The overall survival of the ESCC patients in methylation-dominate group and the methylation-moderate/poor groups.

The survival of the methylation-dominate group and that of the rest two groups were compared in all grades and grades I-III. Abbreviation: ESCC, esophageal squamous cell carcinoma. Source data are provided as a Source Data file.

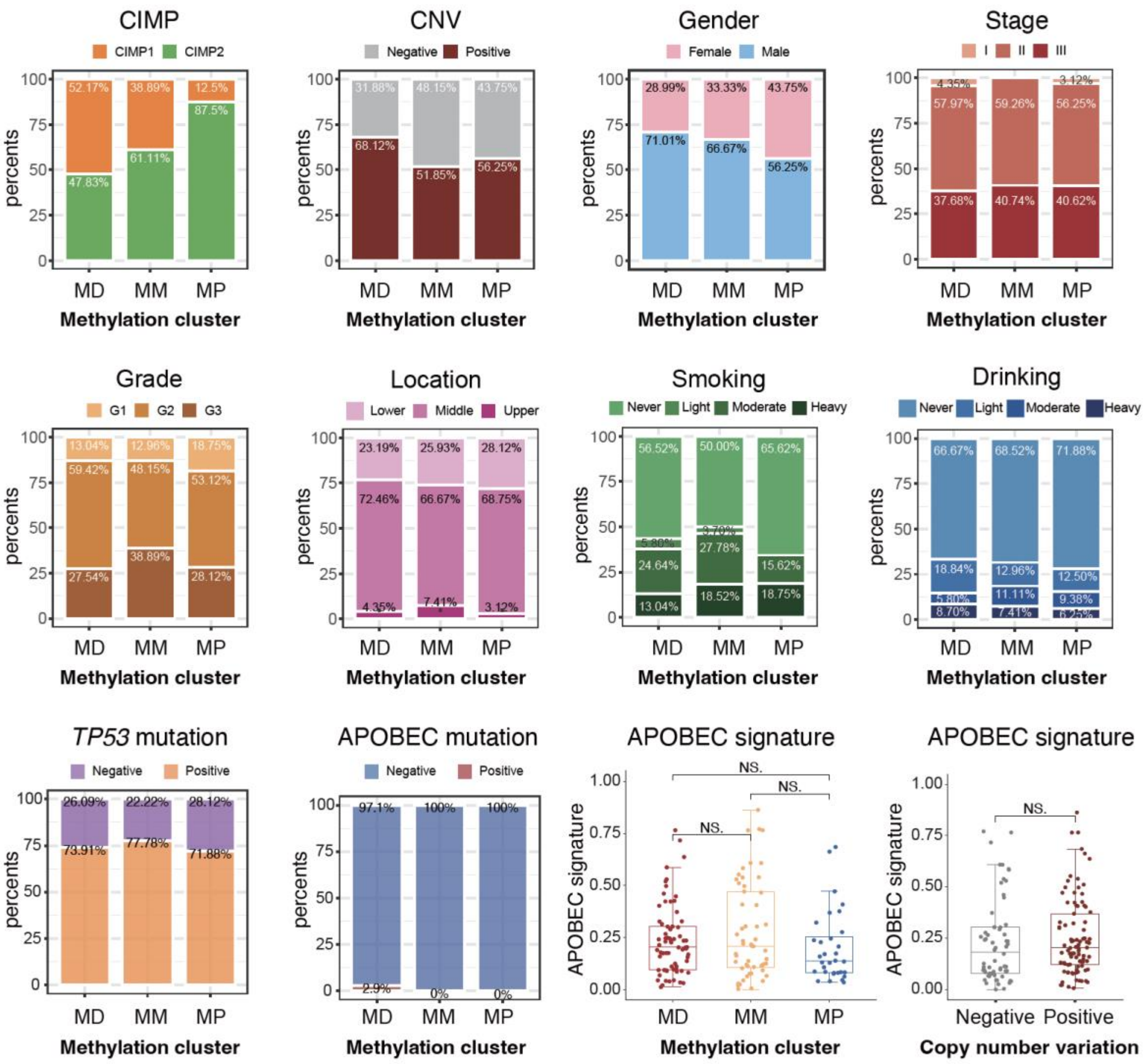

Supplementary Figure 8. The molecular and clinical characteristics of the methylation-dominate, methylation-moderate, and methylation-poor groups.

The proportions of positive CpG island methylator phenotype (CIMP), copy number variant (CNV), gender, stage, grade, location, smoking, drinking, status of *TP53* and APOBEC genes, and the APOBEC mutational signatures in the methylation-dominate (n=69), methylation-moderate (n=54), and methylation-poor groups (n=32). The proportion of the APOBEC mutational signatures was also compared between the ESCC patients with or without CNVs. Data are presented as median values with maximums and minimums. Abbreviation: CIMP, CpG island methylator phenotype; CNV, copy number variant; MD, methylation-dominate group; MM, methylation-moderate group; MP, methylation-poor group; NS, not significant. Source data are provided as a Source Data file.

Supplementary Table 1. Information of the participants.

	**The discovery cohort**		**The external validation cohort**		**The precancerous validation cohort**	
	ESCCs (N=150)	Healthy controls (N=150)	ESCCs (N=30)	Healthy controls (N=30)	IENs (N=50)	Healthy controls (N=50)
Age (mean±SD)	64.24±8.93	59.37±6.50	66.97±8.61	62.00±7.37	61.84±8.95	62.10±6.17
Male (%, n)	72.00% (108/150)	70.67% (106/150)	80.00% (24/30)	80.00% (24/30)	80.00% (40/50)	80.00% (40/50)
Depth (mean±SD)	9.46±1.01	9.75±1.10	9.18±0.60	9.08±0.70	9.32±0.87	9.59±0.92
Stages						
Stage-I (%, n)	60.00% (90/150)	--	30.00% (9/30)	--	--	--
Stage-II (%, n)	14.67% (22/150)	--	20.00% (6/30)	--	--	--
Stage-III (%, n)	14.67% (22/150)	--	50.00% (15/30)	--	--	--
High-grade IEN (%, n)	10.66% (16/150)	--	--	--	76.00% (38/50)	--
Low-grade IEN (%, n)	--	--	--	--	24.00% (12/50)	--

Abbreviation: ESCC, esophageal squamous cell carcinoma; IEN, intraepithelial neoplasia; N, number; SD, standard deviation.

Supplementary Table 2. Performance of ESCC-cfMeth model.

Cutoff value=0.5	Discovery cohort (10-fold cross-validation)	External validation cohort	Precancerous validation cohort
AUC (95%CI)	0.90 (95% CI, 0.87-0.94)	0.89 (95% CI, 0.81-0.98)	0.87 (95% CI, 0.80-0.94)
Sensitivity (%, n)	76.00% (114/150)	80.00% (24/30)	78.00% (39/50)
Specificity (%, n)	88.67% (133/150)	90.00% (27/30)	78.00% (39/50)
PPV (%, n)	87.02% (114/131)	88.89% (24/27)	78.00% (39/50)
NPV (%, n)	78.70% (133/169)	81.82% (27/33)	78.00% (39/50)
Accuracy (%, n)	82.33% (247/300)	85.00% (51/60)	78.00% (39/50)

Abbreviation: ESCC, esophageal squamous cell carcinoma; cfMeth, cell-free DNA methylation; AUC, area under curve; CI, confidence interval; PPV, positive predictive value; NPV, negative predictive value; n, number.

Supplementary Table 3. Performance of combined models.

	**Discovery cohort (10-fold cross-validation)**	**External validation cohort**	**Precancerous validation cohort**
The DMR *plus* CNV model			
AUC (95%CI)	0.98 (95%CI: 0.97-1.00)	0.94 (95%CI: 0.88-1.00)	0.89 (95%CI: 0.83-0.96)
Sensitivity (%, n)	94.67% (142/150)	83.33% (25/30)	58.00% (29/50)
Specificity (%, n)	95.33% (143/150)	96.67% (29/30)	96.00% (48/50)
PPV (%, n)	95.30% (142/149)	96.15% (25/26)	93.55% (29/31)
NPV (%, n)	94.70% (143/151)	85.29% (29/34)	69.57% (48/69)
Accuracy (%, n)	95.00% (285/300)	90.00% (54/60)	77.00% (77/100)
The EMMA model (DMR *plus* CNV and FSR)			
AUC (95%CI)	0.99 (95%CI: 0.98-1.00)	0.95 (95%CI: 0.89-1.00)	0.89 (95%CI: 0.83-0.95)
Sensitivity (%, n)	95.33% (143/150)	86.67% (26/30)	62.00% (31/50)
Specificity (%, n)	95.33% (143/150)	96.67% (29/30)	96.00% (48/50)
PPV (%, n)	95.33% (143/150)	96.30% (26/27)	93.94% (31/33)
NPV (%, n)	95.33% (143/150)	87.88% (29/33)	71.64% (48/67)
Accuracy (%, n)	95.33% (286/300)	91.67% (55/60)	79.00% (79/100)

Abbreviation: DMR, differentially methylated region; CNV, copy number variant; FSR, fragment size ratio; EMMA, expanded multimodal analysis; AUC, area under curve; CI, confidence interval; PPV, positive predictive value; NPV, negative predictive value; n, number.

Supplementary Table 4. The overlapping of the *TP53* and APOBEC genes and the ESCC cfDNA markers.

Gene	Chr	Start	End	Candidate markers
TP53	chr17	7661779	7687550	FSR chr17:5000000-10000000
**The APOBEC Family**				
AICDA	chr12	8602170	8612867	None
APOBEC1	chr12	7649400	7665908	
APOBEC2	chr6	41053304	41064511	
APOBEC4	chr1	183646275	183653316	
APOBEC3A	chr22	38952741	38992778	FSR chr22:35000000-40000000
APOBEC3B	chr22	38982347	38992804	
APOBEC3C	chr22	39014257	39020352	
APOBEC3D	chr22	39021113	39033277	
APOBEC3F	chr22	39040604	39055972	
APOBEC3G	chr22	39077067	39087743	
APOBEC3H	chr22	39097224	39104067	

Abbreviation: APOBEC, apolipoprotein B mRNA-editing enzyme catalytic polypeptide-like; ESCC, esophageal squamous cell carcinoma; cfDNA, cell-free DNA; chr, chromosome; FSR, fragment size ratio.

Reference

1 Loyfer, N. *et al.* A DNA methylation atlas of normal human cell types. *Nature* **613**, 355-364 (2023). https://doi.org:10.1038/s41586-022-05580-6

2 Liu, Z. *et al.* Integrated multi-omics profiling yields a clinically relevant molecular classification for esophageal squamous cell carcinoma. *Cancer Cell* **41**, 181-195.e189 (2023). https://doi.org:10.1016/j.ccell.2022.12.004
