# Supplementary material for: Multimodal analysis of cfDNA methylomes for early detecting esophageal squamous cell carcinoma and precancerous lesions
Source: Nat Commun. 2024 May 2;15:3700. doi: 10.1038/s41467-024-47886-1 (PMC11065998; doi:10.1038/s41467-024-47886-1)
Supplement: Supplementary file 3 — Description of Additional Supplementary Files [file 41467_2024_47886_MOESM3_ESM.pdf]

Description of Additional Supplementary Files

File Name: Supplementary Data 1

Description: Genes in differentially methylated regions of the ESCC-cfMeth model.

File Name: Supplementary Data 2

Description: Genes in regions of copy number variants in ESCC cfDNA.
