# Supplementary material for: Multimodal analysis of cfDNA methylomes for early detecting esophageal squamous cell carcinoma and precancerous lesions
Source: Nat Commun. 2024 May 2;15:3700. doi: 10.1038/s41467-024-47886-1 (PMC11065998; doi:10.1038/s41467-024-47886-1)
Supplement: Supplementary file 6 — Reporting Summary [file 41467_2024_47886_MOESM6_ESM.pdf]

nature portfolio

Corresponding author(s): Zhihua Liu, Jianzhong Su, Hongyan Chen

Last updated by author(s): Mar 10, 2024

Reporting Summary

Nature Portfolio wishes to improve the reproducibility of the work that we publish. This form provides structure for consistency and transparency in reporting. For further information on Nature Portfolio policies, see our Editorial Policies and the Editorial Policy Checklist.

Statistics

For all statistical analyses, confirm that the following items are present in the figure legend, table legend, main text, or Methods section.

n/a	Confirmed	
☐	☒	The exact sample size (*n*) for each experimental group/condition, given as a discrete number and unit of measurement
☐	☒	A statement on whether measurements were taken from distinct samples or whether the same sample was measured repeatedly
☐	☒	The statistical test(s) used AND whether they are one- or two-sided<br>*Only common tests should be described solely by name; describe more complex techniques in the Methods section.*
☐	☒	A description of all covariates tested
☐	☒	A description of any assumptions or corrections, such as tests of normality and adjustment for multiple comparisons
☐	☒	A full description of the statistical parameters including central tendency (e.g. means) or other basic estimates (e.g. regression coefficient) AND variation (e.g. standard deviation) or associated estimates of uncertainty (e.g. confidence intervals)
☐	☒	For null hypothesis testing, the test statistic (e.g. *F*, *t*, *r*) with confidence intervals, effect sizes, degrees of freedom and *P* value noted<br>*Give P values as exact values whenever suitable.*
☒	☐	For Bayesian analysis, information on the choice of priors and Markov chain Monte Carlo settings
☒	☐	For hierarchical and complex designs, identification of the appropriate level for tests and full reporting of outcomes
☒	☐	Estimates of effect sizes (e.g. Cohen's *d*, Pearson's *r*), indicating how they were calculated

Our web collection on statistics for biologists contains articles on many of the points above.

Software and code

Policy information about availability of computer code

Data collection	No software was used for data collection.
Data analysis	Quality control analyses were generated for the raw sequencing data using the FastQC (version 0.11.8, www.bioinformatics.babraham.ac.uk/projects/fastqc/). For tissue samples, trim_galore (version 0.6.0, www.bioinformatics.babraham.ac.uk/projects/trim_galore/) was employed to filter low-quality read data and trim adapters, retaining high-quality data with a quality score greater than 20. For cfDNA samples, the bbduk tool from bbmap was utilized to customize a WGBS adapter library and perform adapter trimming to preserve more fragment information. Subsequently, reads were aligned to the hg38 genome using Bismark (version 22.1) to identify the optimal alignment strategy. The Samtools suite (version 1.9) manipulated alignments in the BAM format. We used bedtools utilities (version 2.30.0) for the comparison, manipulation, and annotation of genomic features in Browser Extensible Data (BED). We performed DMR annotation using ChipSeeker and the TxDb.Hsapiens.UCSC.hg38.knownGene database, which includes human gene transcripts and is accessible via Bioconductor. The Gaussian mixture model from the 'mclust' package (version 5.4.5, https://github.com/Japrin/mclust) was used to cluster the log2Ratio values. The hidden Markov models (HMM) employed the 'HMM' package (version 1.0.1, https://CRAN.R-project.org/package=HMM). The ichorCNA (https://github.com/broadinstitute/ichorCNA) was used as input for cfDNA CNV analysis. The R package 'xcell' (version 1.1.0) was used to predict cell type deconvolution scores. R package 'DESeq2' (version 1.32.0) was utilized in differential expression analysis. GO enrichment analyses were performed by the enrichGO function in the R package 'clusterProfiler' (version 3.10.1). The receiver operating characteristic (ROC) curves were constructed using the 'pROC' package (version 1.18.0). Kaplan-Meier analysis and log-rank tests were conducted using the R-package 'survival' for the overall survival analysis. Bar charts, bar plots, pie charts, line graphs, and other visualizations were generated using the 'ggplot2' package (version 3.4.2). Heatmaps were created using the 'ComplexHeatmap' package (version 2.14.0).<br>The source code in this study was provided in an open-source repository in Github (https://github.com/packageandcode/EMMA).

For manuscripts utilizing custom algorithms or software that are central to the research but not yet described in published literature, software must be made available to editors and reviewers. We strongly encourage code deposition in a community repository (e.g. GitHub). See the Nature Portfolio guidelines for submitting code & software for further information.

Data

Policy information about availability of data

All manuscripts must include a data availability statement. This statement should provide the following information, where applicable:

- Accession codes, unique identifiers, or web links for publicly available datasets
- A description of any restrictions on data availability
- For clinical datasets or third party data, please ensure that the statement adheres to our policy

The WGBS data from 460 cfDNA samples generated in this study have been deposited in the Genome Sequence Archive (GSA) for Human database in the BIG Data Center (http://bigd.big. ac.cn/gsa), Beijing Institute of Genomics (BIG), Chinese Academy of Sciences under accession number HRA006113 (https://ngdc.cncb.ac.cn/gsa-human/browse/HRA006113). The cfDNA WGBS data are available under restricted access for non-profit use, access can be obtained by addressing the corresponding author Zhihua Liu (liuzh@cicams.ac.cn). The multi-omics genome-wide data from tissue samples is available through the GSA database for Human database under accession code HRA003107 (WGS & RNA-seq, https://ngdc.cncb.ac.cn/gsa-human/browse/HRA003107) and HRA003533 (WGBS, https://ngdc.cncb.ac.cn/gsa-human/browse/HRA003533).

Research involving human participants, their data, or biological material

Policy information about studies with human participants or human data. See also policy information about sex, gender (identity/presentation), and sexual orientation and race, ethnicity and racism.

Reporting on sex and gender	Among 155 patients in the ECGEA cohort, the number of male and female patients is 103 and 52, respectively. For cell-free DNA analysis, the proportions of male in ESCC/IEN patients and in healthy controls in the discovery cohort, the external validation cohort, and the pre-cancer validation cohort are 72.00% (108/150), 70.67% (106/150), 80.00% (24/30), 80.00% (24/30), 80.00% (40/50), and 80.00% (40/50), respectively (Extended Data Table 1). The ESCC/IEN patients in the three cohorts were consecutively recruited, and healthy controls were matched by age and gender. These are consistent with the epidemiological characteristics of ESCC.
Reporting on race, ethnicity, or other socially relevant groupings	All the participants involved in this study were of the Chinese population. This study did not analyze race, ethnicity, or other socially relevant categorization variables.
Population characteristics	Population characteristics have been summarized in Extended Data Table 1, including age, gender, and clinical staging.
Recruitment	The ECGEA cohort consisted of 155 patients diagnosed with ESCC between May 2017 and July 2018 at Shanxi Cancer Hospital, China. Samples of ESCC tissue and paired adjacent normal/non-neoplastic tissue were collected before treatment. The discovery cohort comprised 150 consecutive patients diagnosed with ESCC or HGIEN of the esophagus and 150 gender- and age-matched HCs from the CHCAMS between May 2019 and December 2022. All the ESCC patients underwent surgery or endoscopic resection. The external validation cohort enrolled 30 patients with ESCC and 30 gender- and age-matched HCs from the Shanghai Chest Hospital between October 2022 and December 2022. The precancerous validation cohort consisted of 50 patients with esophagus IEN who underwent endoscopic resection and 50 gender- and age-matched HCs from the CHCAMS between August 2022 and February 2023.
Ethics oversight	This study was reviewed and approved by the relevant ethics committees and performed following the Declaration of Helsinki. Collection protocols were approved by the Institutional Review Boards of Shanxi Medical University and Shanxi Cancer Hospital (ECGEA cohort), the institutional review board and the independent ethics committee of the National Cancer Center, Cancer Hospital, Chinese Academy of Medical Sciences (CHCAMS, the discovery cohort and the precancerous validation cohort), and the ethics committee of the Shanghai Chest Hospital (the external validation cohort). All patients provided written informed consent.

Note that full information on the approval of the study protocol must also be provided in the manuscript.

Field-specific reporting

Please select the one below that is the best fit for your research. If you are not sure, read the appropriate sections before making your selection.

☒ Life sciences ☐ Behavioural & social sciences ☐ Ecological, evolutionary & environmental sciences

For a reference copy of the document with all sections, see nature.com/documents/nr-reporting-summary-flat.pdf

Life sciences study design

All studies must disclose on these points even when the disclosure is negative.

Sample size	As there is no pre-specified sample size for the discovery cohort was determined, we consecutively enrolled the patients with esophageal squamous cell carcinoma (ESCC) or esophageal high-grade intraepithelial neoplasia (HGIEN, namely stage-0 ESCC) and gender- and age-matched healthy controls (HCs) at the Cancer Hospital of the Chinese Academy of Medical Sciences and Peking Union Medical College (CHCAMS) between May 2019 and December 2022. Furthermore, we determined the sample size of the validation cohorts using PASS software. The parameters were set as α=0.05, 1-β=0.90, AUC=0.90 (10-fold cross-validation in the discovery cohort), and null hypothesis

	AUC=0.70. The results showed that the number of positive and negative cases was 30. Thus, we enrolled 30 patients with ESCC and 30 matched HCs from the Shanghai Chest Hospital as the external validation cohort. Additionally, to ensure the generalizability of our findings to patients with precancerous lesions, we included a precancerous validation cohort consisting of 50 patients with IEN and 50 matched healthy controls from the CHCAMS.
Data exclusions	No data were excluded in this study.
Replication	The modeling performances were confirmed by the 10-fold cross-validation in the discovery cohort and independent validation in the external cohort.
Randomization	This study was not randomized because this is an observational case-control study without intervention. To avoid selection bias, the patients with esophageal squamous cell carcinoma or esophageal intraepithelial neoplasia and gender- and age-matched healthy controls were consecutively enrolled in each center.
Blinding	Not applicable to this study, as this study is not an interventional study.

Reporting for specific materials, systems and methods

We require information from authors about some types of materials, experimental systems and methods used in many studies. Here, indicate whether each material, system or method listed is relevant to your study. If you are not sure if a list item applies to your research, read the appropriate section before selecting a response.

Materials & experimental systems

n/a	Involved in the study
☒	☐ Antibodies
☒	☐ Eukaryotic cell lines
☒	☐ Palaeontology and archaeology
☒	☐ Animals and other organisms
☒	☐ Clinical data
☒	☐ Dual use research of concern
☒	☐ Plants

Methods

n/a	Involved in the study
☒	☐ ChIP-seq
☒	☐ Flow cytometry
☒	☐ MRI-based neuroimaging

Plants

Seed stocks	*Report on the source of all seed stocks or other plant material used. If applicable, state the seed stock centre and catalogue number. If plant specimens were collected from the field, describe the collection location, date and sampling procedures.*
Novel plant genotypes	*Describe the methods by which all novel plant genotypes were produced. This includes those generated by transgenic approaches, gene editing, chemical/radiation-based mutagenesis and hybridization. For transgenic lines, describe the transformation method, the number of independent lines analyzed and the generation upon which experiments were performed. For gene-edited lines, describe the editor used, the endogenous sequence targeted for editing, the targeting guide RNA sequence (if applicable) and how the editor was applied.*
Authentication	*Describe any authentication procedures for each seed stock used or novel genotype generated. Describe any experiments used to assess the effect of a mutation and, where applicable, how potential secondary effects (e.g. second site T-DNA insertions, mosiacism, off-target gene editing) were examined.*
