# Supplementary material for: Author Correction: Multimodal analysis of cfDNA methylomes for early detecting esophageal squamous cell carcinoma and precancerous lesions
Source: Nat Commun. 2026 Aug 25;17:8972. doi: 10.1038/s41467-026-75462-2 (PMC13507207; doi:10.1038/s41467-026-75462-2)
Supplement: Supplementary file 1 [file 41467_2026_75462_MOESM1_ESM.pdf]

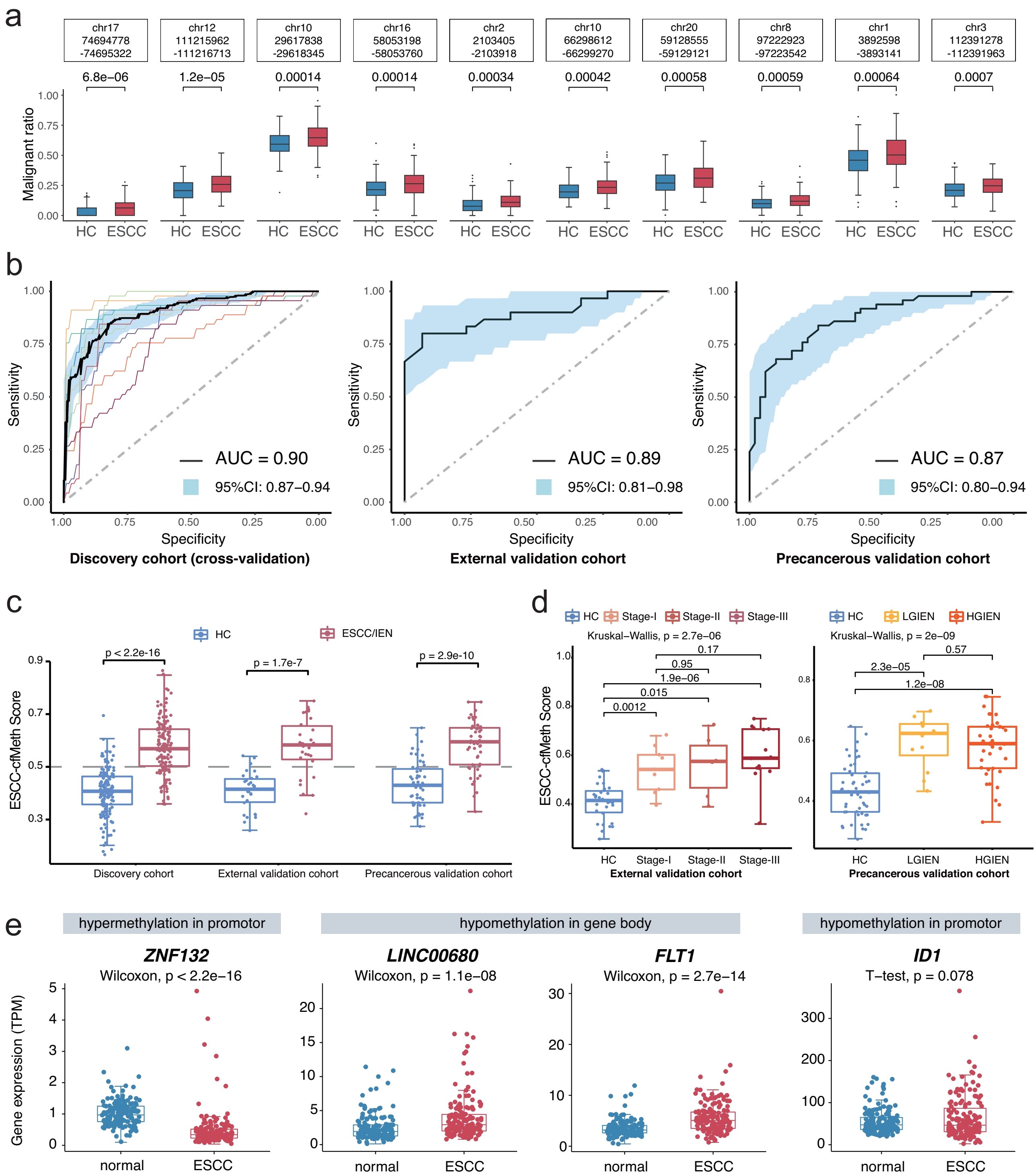
a
chr17 74694778 -74695322
chr12 111215962 -111216713
chr10 29617838 -29618345
chr16 58053198 -58053760
chr2 2103405 -2103918
chr10 66298612 -66299270
chr20 59128555 -59129121
chr8 97222923 -97223542
chr1 3892598 -3893141
chr3 112391278 -112391963
6.8e−06
1.2e−05
0.00014
0.00014
0.00034
0.00042
0.00058
0.00059
0.00064
0.0007
Malignant ratio
HC
ESCC
b
Sensitivity
Specificity
AUC = 0.90
95%CI: 0.87–0.94
Discovery cohort (cross-validation)
AUC = 0.89
95%CI: 0.81–0.98
External validation cohort
AUC = 0.87
95%CI: 0.80–0.94
Precancerous validation cohort
c
HC
ESCC/IEN
ESCC-cfMeth Score
p < 2.2e-16
p = 1.7e-7
p = 2.9e-10
Discovery cohort
External validation cohort
Precancerous validation cohort
d
HC
Stage-I
Stage-II
Stage-III
Kruskal–Wallis, p = 2.7e−06
0.17
0.95
1.9e−06
0.015
0.0012
External validation cohort
LGIEN
HGIEN
Kruskal–Wallis, p = 2e−09
2.3e−05
0.57
1.2e−08
Precancerous validation cohort
e
hypermethylation in promotor
hypomethylation in gene body
hypomethylation in promotor
ZNF132
Wilcoxon, p < 2.2e−16
LINC00680
Wilcoxon, p = 1.1e−08
FLT1
Wilcoxon, p = 2.7e−14
ID1
T−test, p = 0.078
Gene expression (TPM)
normal
ESCC
